# A Systems Immunology Approach to Plasmacytoid Dendritic Cell Function in Cytopathic Virus Infections

**DOI:** 10.1371/journal.ppat.1001017

**Published:** 2010-07-22

**Authors:** Gennady Bocharov, Roland Züst, Luisa Cervantes-Barragan, Tatyana Luzyanina, Egor Chiglintsev, Valery A. Chereshnev, Volker Thiel, Burkhard Ludewig

**Affiliations:** 1 Institute of Numerical Mathematics, Russian Academy of Sciences, Moscow, Russia; 2 Institute of Immunobiology, Kantonal Hospital St. Gallen, St. Gallen, Switzerland; 3 Institute of Mathematical Problems in Biology, Russian Academy of Sciences, Pushchino, Russia; 4 Novosibirsk State University, Novosibirsk, Russia; 5 Institute of Immunology and Physiology, Ural Branch of the Russian Academy of Sciences, Ekaterinburg, Russia; 6 VetSuisse Faculty, University of Zurich, Zurich, Switzerland; Mount Sinai School of Medicine, United States of America

## Abstract

Plasmacytoid dendritic cell (pDC)-mediated protection against cytopathic virus infection involves various molecular, cellular, tissue-scale, and organism-scale events. In order to better understand such multiscale interactions, we have implemented a systems immunology approach focusing on the analysis of the structure, dynamics and operating principles of virus-host interactions which constrain the initial spread of the pathogen. Using high-resolution experimental data sets coming from the well-described mouse hepatitis virus (MHV) model, we first calibrated basic modules including MHV infection of its primary target cells, i.e. pDCs and macrophages (Mφs). These basic building blocks were used to generate and validate an integrative mathematical model for in vivo infection dynamics. Parameter estimation for the system indicated that on a per capita basis, one infected pDC secretes sufficient type I IFN to protect 10^3^ to 10^4^ Mφs from cytopathic viral infection. This extremely high protective capacity of pDCs secures the spleen's capability to function as a ‘sink’ for the virus produced in peripheral organs such as the liver. Furthermore, our results suggest that the pDC population in spleen ensures a robust protection against virus variants which substantially down-modulate IFN secretion. However, the ability of pDCs to protect against severe disease caused by virus variants exhibiting an enhanced liver tropism and higher replication rates appears to be rather limited. Taken together, this systems immunology analysis suggests that antiviral therapy against cytopathic viruses should primarily limit viral replication within peripheral target organs.

## Author Summary

Human infections with highly virulent viruses, such as 1918 influenza or SARS-coronavirus, represent major threats to public health. The initial innate immune responses to such viruses have to restrict virus spread before the adaptive immune responses fully develop. Therefore, it is of fundamental practical importance to understand the robustness and fragility of the early protection against such virus infections mediated by the type I interferon (IFN) response. Because of the inherent complexity of the virus-host system, we have used mathematical modeling to predict the sensitivity of the kinetics and severity of infection to variations in virus and host parameters. Our results suggest that the spleen represents a robust sink system for systemic virus infection and that this system is able to cope with substantial variations in IFN secretion and virus production. However, the system is very fragile to only minor increases in the virus growth rate in peripheral tissues. Collectively, the mathematical approach described in this study allows us to identify the most robust virus and host parameters during early cytopathic virus infection and can serve as a paradigm for systems immunology analyses of multiscale virus-host interaction of many life-threatening cytopathic virus infections.

## Introduction

Protection against life-threatening infections is a major function of the immune system. The systems biology view of the induction of the protective immune responses suggests that the kinetics of innate immune responses critically impinge on the development of pathogen-specific adaptive immune responses [1]. The major services provided by cells of the innate system located in secondary lymphoid organs (SLO) are (i) an early sensing of pathogen-associated molecular patterns (ii) the reduction of pathogen spread throughout the host by capturing pathogens, and (iii) the sustained stimulation of the adaptive responses over sufficient periods of time [2]. To mediate these challenging functions of pathogen capturing and containment, and long-lasting antigen presentation, efficient cell protection mechanisms are needed, especially in the case of cytopathic virus infections.

Plasmacytoid dendritic cells (pDCs) are a CD11c^low^ DC subset that is characterized by a particular set of phenotypic markers and special functional properties [3], [4]. One of the major functional characteristics of pDCs is the expression of pathogen recognition receptors, such as Toll-like receptor (TLR)-7 and -9, which endow these cells with the ability to rapidly produce large amounts of type I interferons (IFNs) following encounter with RNA or DNA viruses [5]. Hence, by providing a first wave of antiviral IFN, pDCs immediately limit viral spread and set the stage for antigen-specific immune responses.

**Figure 1:**
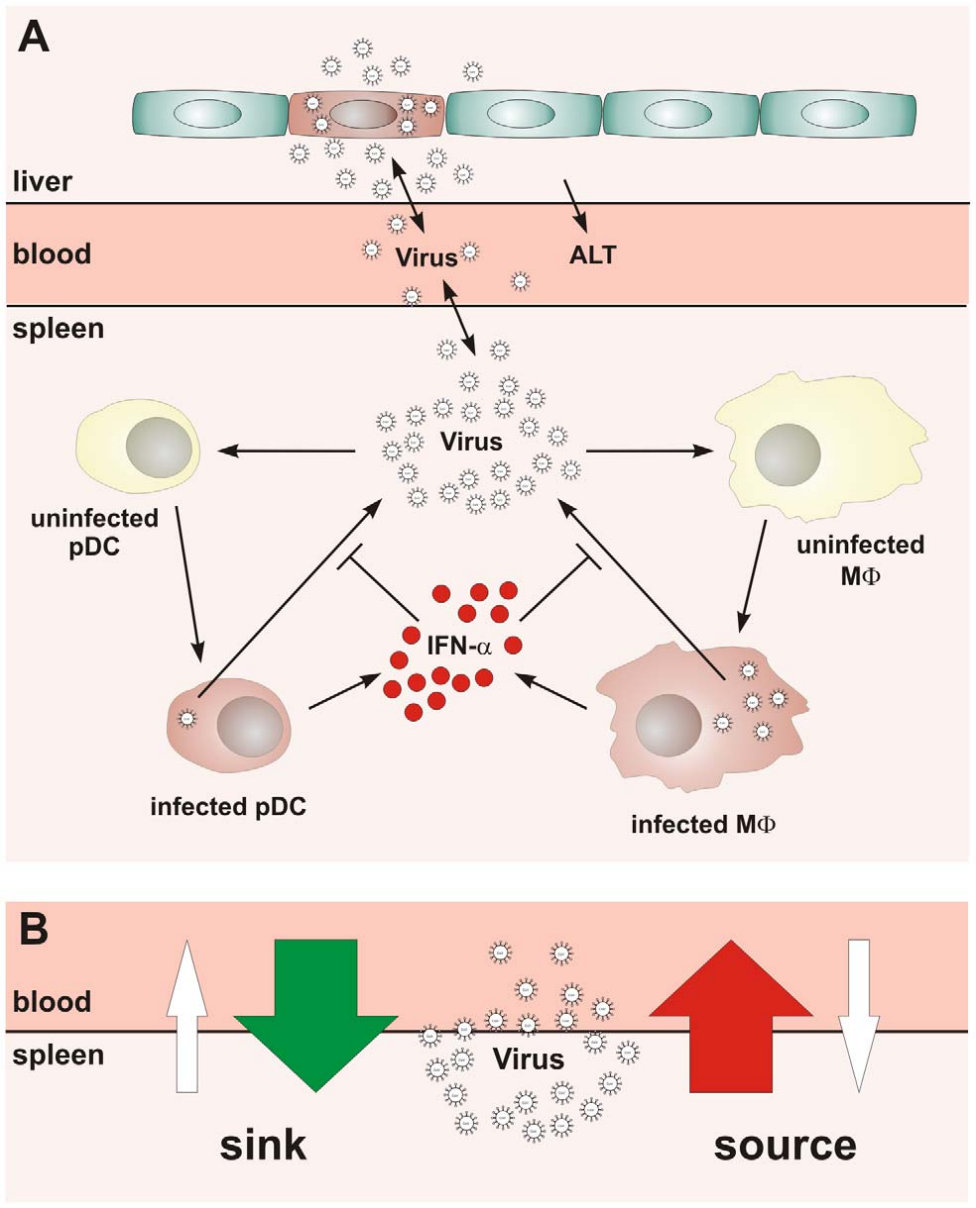
Conceptual scheme of type I IFN responses during cytopathic coronavirus infection. (A) Systemic view of the processes determining the early kinetics of mouse hepatitis virus (MHV) infection. (B) Schematic depiction of the ‘sink’ versus ‘source’ function of spleen in coronavirus infection.

The mouse hepatitis virus (MHV) infection represents a well-understood paradigmatic system for the analysis of type I IFN responses. MHV is a member of the *Coronaviridae* family that harbor a number of viruses causing severe diseases in animals and humans, such as acute hepatitis, encephalitis, infectious bronchitis, lethal infectious peritonitis, and the severe acute respiratory syndrome (SARS) [6], [7]. In systemic MHV infection, spleen and liver represent major target organs [8], and primarily hematopoietic cell-derived type I IFN controls viral replication and virus-induced liver disease [9]. We could recently show that pDCs are the major cell population generating IFN-α during the initial phase of mouse coronavirus infection [8]. Importantly, mainly macrophages (Mφ) and, to a lesser extent conventional DCs, respond most efficiently to the pDC-derived type I IFN and thereby secure containment of MHV within SLOs [10]. Thus, the type I IFN-mediated crosstalk between pDCs and Mφs represents an essential cellular pathway for the protection against MHV-induced liver disease. In system biology terms, MHV infection triggers a complex array of processes at different biological scales such as protein expression, cellular migration, or pathological organ damage. To focus on the front edge of the virus-host interaction, the present analysis specifically addresses the early dynamics (i.e. the first 48 h) of the type I IFN response to MHV since this is decisive for the outcome of the infection. The reductionist's view of the most essential processes underlying the early systemic dynamics of MHV infection, liver pathology and the first wave of type I IFN production is summarized in Figure 1A.

Our studies on the role of pDCs in establishing the type I IFN-mediated protection of Mφs against cytopathic MHV infection suggested that the spleen may function as a ‘sink’ contributing to the elimination of the virus from the system. Under the condition of pDC-deficiency or lack of type I IFN responsiveness of Mφs, a severe disease is observed [8], [10] indicating that the operation of the spleen might switch from a ‘virus sink’ to a ‘virus source’ mode. This bi-modal function, i.e. the ability of the spleen to either eliminate or disseminate the virus, is outlined in Figure 1B. The switch between the two modes most likely depends on the number of pDCs in spleen, their activation status, the dose of infection, and the kinetics of virus spread. Thus, for an improved understanding of pDC function in cytopathic virus infection, it is of fundamental importance to determine the robustness and fragility of the early type I IFN response within SLOs in relation to the ability of the virus to counteract the IFN system and to replicate and cause severe disease in peripheral tissues. Because of the inherent complexity of the virus-host system, experimental approaches to examine the dynamical aspects of such multiscale interactions are limited. Therefore, we have used mathematical modeling in conjunction with high-resolution experimental data to predict kinetics and severity of infection in relation to variations in virus and host parameters. Our results suggest that the spleen represents a robust sink system for cytopathic virus infection able to cope with substantial variations of the IFN secretion and virus production in the spleen. However, the system is very fragile to minor increases in the virus growth rate in peripheral tissues.

## Results

To describe quantitatively the structure, dynamics and the operating principles that permit pDCs to initially shield the host against an overwhelming spread of the cytopathic MHV infection, we followed a systems biology approach as outlined previously [11], [12]. First, we decomposed the system dynamics into a set of ‘elementary’, well-documented processes such as the virus replication, target cell turnover and IFN-α decay, as well as the production of virus and IFN-α by infected cells (Figure 1A). This allowed us to estimate the individual decay rates, the virus-target cell interaction parameters and the protective effect of IFN-α. Once these elementary modules of virus-target cell interactions were calibrated, we used them as building blocks to set up an integrated mathematical model of pDC-mediated type I IFN responses against MHV infection in mice.

### Modeling the in vitro kinetics of MHV infection

**Figure 2:**
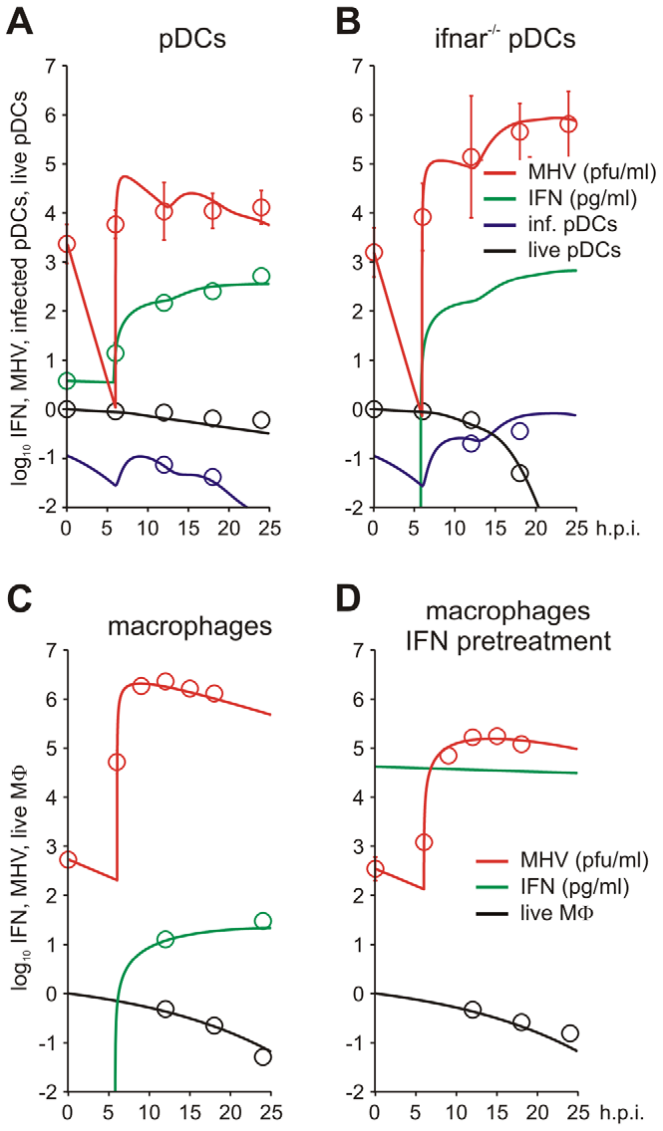
MHV infection and IFN response kinetics in vitro. Experimental data (open symbols) represent the geometric mean ±SEM. Virus titers (red), IFN-α concentration (green) and the fraction of live cells out of the total initial target cell number (black) were used to calibrate the model. (A) In vitro MHV infection of pDCs (MOI = 1). Fraction of infected cells in the population of live cells is shown in blue. (B) MHV infection in pDCs derived from *ifnar* ^−/−^ mice. Fraction of infected cells in the population of live cells is shown in blue. Experimental validation had been performed using infection of pDCs with EGFP-recombinant MHV (blue circles). (C) MHV replication in wild-type Mφs. The data on the amount of secreted IFN-α were used to validate the calibrated model (green circles). (D) Effect of IFN pre-treatment (500 IU of IFN-α, green line) on MHV replication in wt Mφs.

To estimate the kinetic parameters of MHV-pDC interaction, we used in vitro data on MHV infection of bone marrow-derived pDCs as described previously [8]. The data set characterizes the response of pDCs infection with MHV at a multiplicity of infection (MOI) of 1 (Figure 2A). To delineate a quantitative effect of IFN-α on virus production, additional data from similar experiments conducted with pDCs from mice deficient for the type I IFN receptor (*ifnar* ^−/−^) were used (Figure 2B). In addition to the MHV/IFN-α data, we considered data on survival kinetics of MHV-infected pDCs from wt and *ifnar* ^−/−^ mice generated independently in a separate series of experiments. The MHV-pDC interaction parameters appearing in the basic model of the type I IFN response (described in Materials and Methods) were estimated by fitting simultaneously the data sets on wt and *ifnar* ^−/−^ cells. The maximum likelihood approach for the log-transformed data was used to quantify the model parameters with the resulting best-fit description of the data by the model shown in Figure 2A and 2B. The resulting calibrated model for the in vitro pDC response to MHV was further validated by comparing its predictions with in vitro infection at an MOI of 0.1 and 0.01 (Figure S1) and also, by determining the fraction of infected cells deduced from experimental data sets using an enhanced green fluorescence protein (EGFP) expressing recombinant MHV [10] (Figure 2A and B). The parameter values summarized in Table 1 provide additional insight into the ‘numbers game’ between the virus and pDCs: (i) the average MHV secretion rate of infected pDCs is rather low with ∼1.7 pfu cell^−1^ h^−1^, (ii) the IFN-α level required for 2-fold inhibition of MHV production is about 46 pg/ml, (iii) the average secretion rate of IFN-α per infected pDC is ∼4.4*10^−4^ pg h^−1^ or, equivalently, ∼15586 molecules h^−1^. The latter estimate takes into account that the molecular weight of IFN-α is about 17000 atomic mass units (a.u.) and 1 a.u. = 1.67×10^−24^ *g*.

**Table 1:** Best-fit parameter values for in vitro and in vivo MHV infection of pDC and Mφ and the corresponding 95% confidence intervals.

Biological parameter; notation (units)	pDC best-fit estimates [95% CI]	Mφ best-fit estimates [95% CI]
Virus production rate, 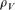 (*pfu/cell/h*)	1.7 [0.62, 5.5]	36.7 [18, 220]
Type I IFN production rate, 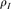 (*pg/cell/h*)	4.4×10^−4^ [1.3×10^−4^, 1.6×10^−3^]	3.0×10^−6^/1.0×10^−6^ * [1.2×10^−6^, 1.9×10^−5^]
The threshold for 50% reduction of virus production rate by type I IFN, 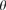 (*pg/ml*)	45.8 [26, 80]	0.09/0.97* [0.007, 0.7]
Infection rate of target cells, 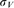 (*cell/pfu/h*)	1.3×10^−6^ [7.0×10^−7^, 2.8×10^−6^]	5.4×10^−6^/0.9×10^−7^ * [1.7×10^−6^, >10^−4^]
Initial fraction of infected cells for MOI = 1; *C_V_*(0)	0.11 [0.029, 0.26]	1.0
Virus production delay; 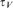 (*h*)	5.96 [5.88, 5.98]	5.99 [5.98, 6.0]
Type I IFN production delay; 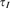 (*h*)	5.77 [5.22, 5.93]	5.8
Gompertz death rate parameters for infected cells, *d_0CV_* (*1/h*) *& k_CV_* (*1/h*)	0.2 & 0.087 [0.015, 6.0] & [0.029, 0.19]	0.049 & 0.057 [0.024, 0.11] & [0.012, 0.11]

*Refined by in vivo data.

To identify the parameters of MHV infection and type I IFN-mediated protection of Mφs, we considered experimental data sets that were generated using a broad spectrum of IFN treatment conditions [10], [13]. These data sets included (i) the early kinetics of MHV replication in Mφs at MOI = 1 (Figure 2C) and 0.0001 (Figure S2), (ii) Mφ infection (MOI = 1) after treatment with 500 *IUnits* (1 IU≅8.333 *pg*) of recombinant IFN-α (Figure 2D) and (iii) Mφ infection (MOI = 1) after pre-treatment with pDCs derived supernatant containing 500, 200, 50 and 10 pg/ml of IFN-α (Figure S3). The core data set using MOI = 1 was supplemented by Mφ survival data generated as described previously [10]. As shown in Figure 2C and D, the experimental data for MHV infection kinetics (MOI = 1) in wt Mφs and after IFN treatment, are in close agreement with the model prediction. The essential parameters (Table 1) suggest that (i) MHV production by a single Mφ is with 37 pfu h^−1^ much larger than that of pDCs, (ii) the concentration of IFN-α required for 2-fold inhibition of MHV production is about 0.1 pg/ml, and (iii) the per cell secretion rate of IFN-α is about 100-times smaller in Mφs (3×10^−6^ pg h^−1^ or equivalently, 106 molecules h^−1^) compared to pDCs. The calibrated modules for the in vitro infection of pDCs and Mφs thus provide valuable basic building blocks that allowed to proceed with the modeling of early kinetics (0–48 hours) of MHV growth and the IFN response in vivo.

### Modeling systemic MHV infection

To further validate the calibrated modules, we considered an experimental in vitro system mimicking ‘in vitro spleen infection’. To this end, we first determined the cellular composition of spleen in terms of pDC and Mφ population sizes during early MHV infection. At the beginning of infection with 5×10^3^ pfu, the geometric means for pDCs and Mφs were 6.6×10^5^ cells and 5.2×10^6^ cells, respectively (relative variation ∼10%), and increased about two-fold by 36 hours following infection. Here, we considered intermediate values, i.e. the ones observed 18 hours post infection, so that 7×10^5^ pDCs and 6×10^6^ Mφs were used to model the infection dynamics in spleen. To evaluate the qualitative consistency of the in vitro parameter estimates with the actually observed phenotype of MHV infection in vivo, we modeled the infections of the mixture of the above numbers of pDCs and Mφs with increasing virus doses (5×10^1^, 5×10^3^, 5×10^5^ pfu). As shown in Figure S4, the model consistently predicts that the virus growth is robustly controlled and that the extent of the activation of the type I IFN response depends on the virus kinetics.

**Figure 3:**
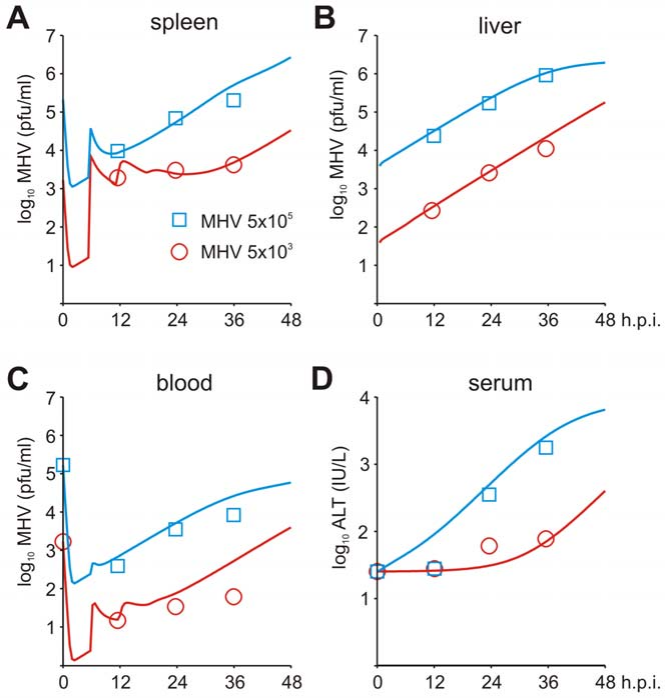
Systemic dynamics of MHV infection in mice. Experimental determination of virus kinetics in (A) spleen, (B) liver and (C) blood, and (D) ALT levels in serum following i.v. infection with MHV at intermediate (5×10^3^ pfu) and high (5×10^5^ pfu) doses (symbols). The mathematical model (solid lines) integrates the detailed virus-target cell kinetics and IFN response in spleen with compartmental virus dynamics in blood and liver.

To set up the mathematical model for systemic MHV infection in mice, we proceeded in stages. First, we estimated the virus transfer rates between blood, spleen and liver together with the rate of alanine aminotransferase (ALT) increase using the compartmental model described in the Materials and Methods section, equations (5)–(8). A reference data set characterizing the MHV growth in spleen, blood and liver and serum ALT kinetics after intravenous (i.v.) infection with 5×10^3^ pfu and 5×10^5^ pfu as shown in Figure 3A–D was used. Next, we integrated the description of MHV infection in spleen (given by equations (9)) with virus compartmental dynamics in the liver and blood to formulate the systemic model of MHV infection specified by the set of delay-differential equations (6)–(9). It is most likely that both the splenic microarchitecture and the trafficking of the virus between organ compartments have an effect on the kinetics of MHV infection of splenic target cells as compared to the in vitro system. The compartmental model parameters listed in Table 2 were estimated via the maximum likelihood approach constrained by a detailed description of MHV interaction with the populations of pDCs and Mφs in spleen. To accommodate for the observed differences between the in vitro and in vivo systems, we set out to refine some of the spleen model parameters to accurately describe the systemic virus data (Figure 3). The number of reliably identifiable parameters in the mathematical model is limited by the amount and quality of the corresponding sets of experimental data which are available. To move from in vitro to in vivo MHV infection the following considerations were taken into account. First, the morphology of spleen is drastically different from the in vitro cell suspensions, which directly implies that the rate of target cell infection might differ. The second factor is the spatial location of Mφs versus pDCs in spleen. Finally, the number of Mφs in spleen is about 10-fold larger than the pDC population, so that the in vitro estimated infection rate of Mφs would lead to an overwhelming virus growth in spleen before enough pDCs get activated to produce a protective amount of type I IFN. Therefore, the kinetics of Mφ infection in spleen has been considered in the first instance. The above reasoning in conjunction with the parsimony principle and numerous trials to fit the in vivo data with different selections of adjusted parameters led us to conclude that a minimal set of three parameters ensured a consistent fitting of the vivo infection data: the infection rate 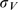 (reduced by 60-fold), the 50% inhibition threshold 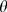 (increased by 10-fold) and the IFN secretion rate 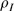 (reduced by 3 fold) for Mφs (Table 1). Taken together, the stepwise developed mathematical model, comprised of calibrated, refined and validated elementary modules, tightly fits the observed in vivo kinetics of MHV infection, and thus, provides a quantitative computational tool to assess the sensitivity of MHV infection dynamics to variations in the basic parameters of virus-host interactions.

**Table 2:** Parameter estimates for a systemic spread of MHV in the anatomical compartments and 99% confidence intervals.

Biological parameter, notation (*units*)	Best-fit estimates [99% CI]
Virus transfer rate: blood to spleen, μ*_BS_* (*1/h*)	3.46 [1.14, 88]
Virus transfer rate: blood to liver, μ*_BL_* (*1/h*)	0.018 [0.89×10^−3^, 1.55]
Virus transfer rate: spleen to blood, μ*_SB_* (*1/h*)	0.91 [0, 1.5]
Virus transfer rate: liver to blood, μ*_LB_* (*1/h*)	0.61 [0.14, 5.7]
Virus elimination from blood, μ*_BO_* (*1/h*)	1.22 [0, 94]
Virus growth rate in liver, β*_L_* (*pfu/ml/h*)	0.78 [0.32, 5.8]
Carrying capacity of the liver, *K_L_* (*pfu/ml*)	10^7^ [4.6×10^5^, >1.5×10^7^]
Rate constant of ALT release into blood, ρ*_A_* (*IU/L*)	0.68×10^−3^ [0.28×10^−4^, 0.01]
Decay rate of ALT, *d_A_* (*1/h*)	0.16 [0.01, 1]
Physiological level of ALT, A^*^ (*IU/L*)	25

### Effect of pDC number and activation status on early antiviral protection

**Figure 4:**
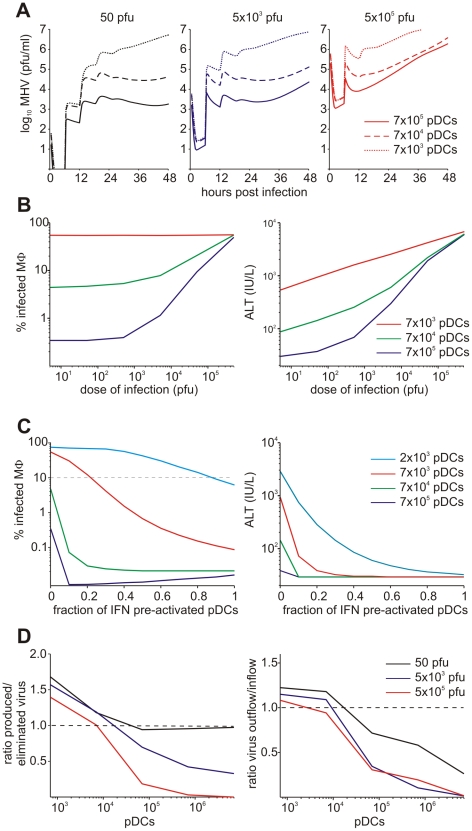
Effects of variation in pDC number and activation status. (A) Virus kinetics in spleen following infection with low (5×10^1^ pfu), intermediate (5×10^3^ pfu) and high (5×10^5^ pfu) dose infections under conditions of varying pDC numbers per spleen. (B) Protection of splenic Mφs determined as percentage of infected Mφs (left panel) and disease severity determined as ALT levels serum (right panel) at 48 h post infection plotted against different doses of infection. For physiological infection doses, the normal number of pDCs (blue line) and 10-fold depleted population (green line) ensure protection of more than 90% of Mφs. Further decrease of pDC population to 7×10^3^ cells is associated with large-scale infection of Mφs (red line). (C) Effect of pre-activation of pDCs in spleen on protection of splenic Mφs determined as percentage of infected cells (left panel) and disease severity determined as ALT levels in serum (right panel) at 48 h post infection (i.v. infection with 50 pfu). Numbers of pDCs per spleen were varied as indicated. (D) Effect of the number of pDCs in spleen on the ratio of locally produced versus eliminated virus (left panel) and on sink versus source function (right panel) after low (5×10^1^ pfu), intermediate (5×10^3^ pfu) and high (5×10^5^ pfu) dose infection under conditions of varying pDC numbers per spleen.

As a first step in the analytical modeling process, we examined the effect of pDC numbers and activation status on the protection of Mφs in spleen and the prevention of severe liver disease. As readout, i.e. the prediction of pDC performance criteria, we considered the maximum fraction of infected Mφs in spleen and the peak level of serum ALT during the first 48 hours post i.v. infection with various doses of MHV. As shown in Figure 4A, the decrease of the pDC population in spleen by 10-fold results in increased virus titers but still keeps virus growth under control. However, further depletion of pDCs leads to an overwhelming virus growth. Mφs in spleen represent about a 10-fold larger population of cells able to secrete MHV at a rate that is 10-times higher than pDCs. Therefore, protection of Mφs against the infection represents an important task that pDCs have to ensure. Indeed, antibody-mediated depletion of pDCs considerably increased infection of splenic Mφs (Figure S5). Figure 4B (left panel) shows the quantitative model predictions of how the fraction of infected Mφs in spleen depends on the number of pDCs and the dose of infection. Ten-fold reduction of pDCs in spleen still ensures that more than 90% of Mφs remain uninfected for low to intermediate infection doses. However, a further decrease of the pDC population breaks their ability to keep the number of infected Mφs below 10%. Because there is an inherent delay in activation of pDCs before the type I IFN secretion starts, we modeled the situation when a certain fraction of splenic pDCs is pre-activated at the start of the infection. Figure 4C (left panel) shows the predicted dependence of the infected Mφs on the number of pre-activated pDCs for MHV infection with 50 pfu. The results allowed us to quantify the upper limit for the protective capacity (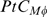) of pDCs, if we define it as the ability to protect 90% of Mφs against infection. As few as 2000 activated pDCs suffice to protect 6×10^6^ Mφs, which leads to the estimate of 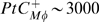 Mφ per pDC. To clarify how the pDCs in spleen contribute to control against severe disease, we evaluated the peak ALT level for i.v. infections with different MHV doses and different pDC numbers (Figure 4B, right panel). If we define the ALT threshold for protection against severe disease to be 10^3^ IU/L, then the host is protected against infection with physiological doses when the number of pDCs in spleen is unchanged (7×10^5^) or 10-fold reduced. The protection is lost if spleen contains only 7×10^3^ pDCs and the dose of infection is larger than 100 pfu. This suggests that the protection unit of pDCs (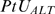) required to prevent severe disease after low dose infection is around 7×10^3^ pDCs. Pre-activation of pDCs leads to a more efficient control of the infection-associated disease as shown in Figure 4C (right panel). The reduction in the total number of non-activated splenic pDCs strongly affects the severity of disease. However, rather modest pre-activation of as few as 200 pDCs leads to a reduction of peak ALT below the threshold of severe disease (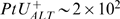).

SLOs function to protect again severe disease by eliminating the virus from the system. To examine how the sink function of spleen depends on the availability of pDCs, we calculated the ratio of the number of viruses produced locally in spleen versus virus eliminated via trapping by target cells. The results summarized in Figure 4D show that the capacity of the spleen to eliminate the virus depends on the number of pDCs. Furthermore, the extent of the sink function depends on the dose of infection, i.e. high dose infection leads to a full activation of the capacity of spleen to work as a sink for the virus. Overall, the spleen preserves its sink function as long as the pDC population is above 10^4^ cells.

### Effects of IFN secretion and virus growth rates

**Figure 5:**
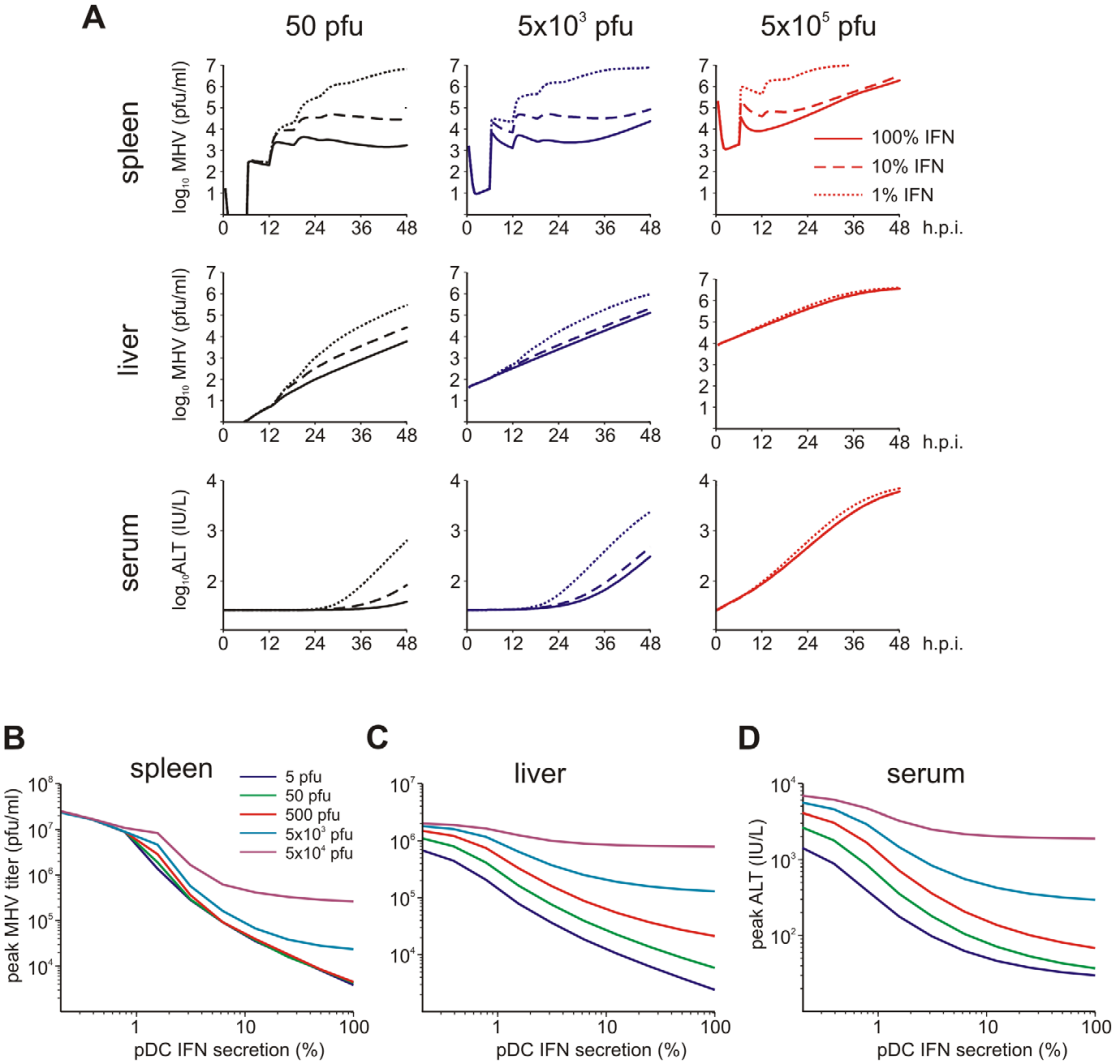
Effect of type I IFN secretion rate on protection against disease. (A) Kinetics of MHV in spleen (upper row) and liver (middle row), and impact on disease severity (bottom row) for normal (solid line), 10-fold reduced (broken line) or 100-fold (dotted line) reduced IFN secretion rate by pDCs after low (5×10^1^ pfu), intermediate (5×10^3^ pfu) and high (5×10^5^ pfu) dose infection. (B) Dependence of peak virus titers in spleen (B) and liver (C) after infection with the indicated doses on the relative reduction rate of IFN secretion rate by pDCs at 48 h post infection (i.v. infection with indicated doses). (D) Disease severity determined as ALT levels in serum at 48 h post infection (i.v. infection with indicated doses) depending on the relative reduction rate of IFN secretion rate by pDCs.

Viruses have evolved various mechanisms to reduce the efficacy of innate immune mechanisms [14]. Therefore, we examined a situation of virus-mediated inhibition of type I IFN synthesis by pDCs. Figure 5A shows the overall kinetics of the virus in spleen and liver together with serum ALT level after low-, intermediate- and high dose i.v. infections of hosts with either normal IFN secretion rate 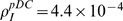 pg cell^−1^ h^−1^, or 10- to 100-fold reduction. Reduced IFN production by pDCs has a stronger impact on virus kinetics in spleen (Figure 5A, upper row) but leads to minor perturbations of virus growth in the liver (Figure 5A, middle row), or ALT levels in serum (Figure 5A, bottom row). To quantitatively estimate the robustness of the pDC-mediated protection for spleen and liver we examined at a higher resolution the effect of reduced IFN synthesis on the peak viral load with infection doses ranging from 5 to 5×10^4^ pfu. Figure 5B shows that the spleen is well-protected, i.e. the peak virus titer stays below 10^5^ pfu/ml. However, once the IFN secretion rate is reduced by 100-fold, the virus infection is out of control as manifested by maximum titers of ∼10^7^ pfu/ml for all doses. In contrast to the spleen, the peak virus titer in the liver increases with higher virus doses and reduced type I IFN secretion rates (Figure 5C). Likewise, the severity of disease, characterized by the peak ALT levels, depends on the dose of infection and the IFN secretion rate by pDCs in a way similar to the virus titer in liver (Figure 5D). If we define a severe disease by ALT levels above 10^3^ IU/L, then 7×10^5^ pDCs in spleen keep the host protected during the first two days of infection with virus doses up to 500 pfu even when the IFN secretion rate drops down to 1% of its normal value.

**Figure 6:**
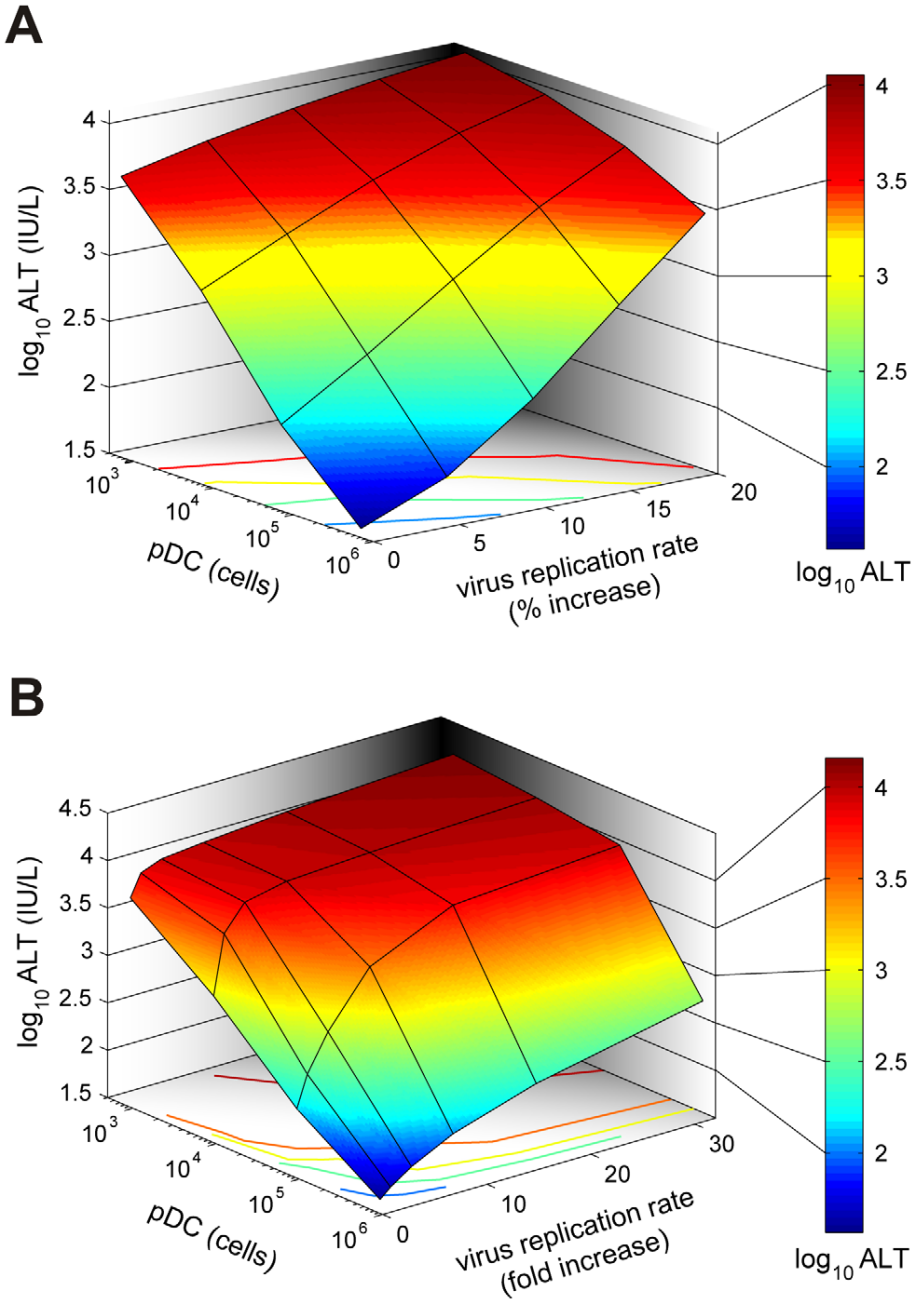
Effect of virus growth rates on pDC-mediated protection against disease. (A) Sensitivity of the disease severity to variations in pDC numbers (cells per spleen) and the global increase of viral replication rate in the liver (% increase). Disease severity is determined as peak ALT levels in serum within 48 h post infection following i.v. infection with 50 pfu. (B) Determination of the system's robustness against disease with respect to variations in pDC numbers (cells per spleen) and increasing viral replication rates restricted to Mφs in the spleen (Note: fold increase).

Viruses can acquire mutations that result in a faster replication in target cells and a high-virulence phenotype [15], [16]. A recently published study on cytopathic influenza A virus infection [15] provides quantitative details of the scale of virulence-enhancing mutations and the resulting increase in virus growth rate. It follows from the analysis of these virus growth data that the difference in the intrinsic growth rate is about 30%. With this estimate as a reference value, we used the mathematical model of MHV infection to evaluate the limits of protection against severe disease for increasing virus replication rates. Since various MHV strains display significant differences in their ability to replicate in different organ systems [17], two complementary scenarios were considered: the increase in virus growth rate in the peripheral organs (liver) versus SLOs (spleen). Figure 6A shows that pDCs in spleen provide very limited protection against severe disease for faster replicating strains of the virus. Indeed, only a 15% increase in the growth rate of MHV in the liver leads to infection with ALT levels rising to 10^3^ IU/L within two days. The decrease of pDC numbers in spleen makes the situation more fragile to even smaller increases in the virus growth rate. The contour lines shown in Figure 6A are the curves along which the value of ALT in serum at 48 h post infection remains the constant. The quantitative analysis of the contour lines slope suggests that 1% increase in the replication rate of the virus in the liver requires about 50% increase in the initial pDCs number in the spleen for the ALT level to have the same particular value. On the contrary, pDCs provide a robust protection against severe disease when the virulence-enhancing mutation leads to faster replication only in target cells located in spleen (Figure 6B), i.e. splenic pDCs protect against severe disease for up to 30-fold increase in the viral replication rate in splenic Mφs. Taken together, these analyses indicate that the spleen represents a robust sink system able to cope with substantially enhanced virus production as long as this gain of viral fitness remains restricted to this SLO.

## Discussion

An important hallmark of the innate immune response during cytopathic virus infection is the ability of Mφs to contain viral particles in SLOs. For example, Mφs in the marginal sinuses of lymph nodes, capture viral particles from the incoming lymph stream [18], and marginal zone Mφs in spleen bind viruses decorated by complement and natural antibodies and reduce thereby dissemination of viruses to peripheral organs [19]. Coronaviruses can suppress early type I IFN responses in particular cell types including Mφs [14], [20], thus leaving these cells vulnerable to the cytopathic effects of the viral infection. Such blocking of innate type I IFN induction has not been observed for MHV and SARS coronavirus infection of mouse and human pDCs, respectively [8]. Likewise, human pDCs seem not to be sensitive to the inhibitory effects of the potent IFN antagonist NS1 of influenza virus [21]. Hence, pDCs are not only specialized for immediate viral recognition and type I IFN production through TLR-7 and -9 expression and the constitutive expression of IRF-7 [5], they probably also exhibit unique counter-measures against viral IFN inhibitors. The interplay between pDCs and Mφs is thus a critical cellular axis for the preservation of SLO integrity during early cytopathic virus infection. The systems immunology approach presented in this study provides a better understanding of the robustness and fragility of the pDC-mediated protection of Mφs and eventually, the host against cytopathic virus infection. We implemented a genuine systems methodology using a building block approach in which the elementary ‘modules of response’ were calibrated using in vitro data. In addition, in vivo observations were used to further estimate essential parameters which combine the modules in a ‘closed’ system. A key to the development of this predictive mechanism-based modular mathematical model were model-driven experimental studies which provided comprehensive data sets for an accurate and reliable quantification of model parameters and validation of the model predictions. Using this approach, we identified the limits of the spleen's capability to function as a sink for the virus produced in a peripheral target organ. The robust sink function of the spleen is guaranteed by the high protective capacity of single pDCs which protect 10^3^ to 10^4^ Mφs from cytopathic viral infection. Furthermore, we determined the minimal protective unit of pre-activated pDCs in spleen to be around 200 cells which can rescue the host from severe disease. The presented results suggest that the splenic sink function remains operational as long as viral mutations do not permit accelerated growth in peripheral tissues.

Our model suggests that maintaining the sink function of SLOs is one of the major functions of pDCs. This notion is supported by findings on the life cycle of pDCs, namely that after development in the bone marrow, pDCs cells enter the blood circulation, and subsequently home to SLOs [22]–[25]. The rapid accumulation of pDCs in lymph nodes following exposition to inflammatory stimuli [26], [27] further corroborates that SLOs are – at least in the very early phase of an infection – the most important compartment of pDC activity. Potential effects of pDCs migrating to non-lymphoid organs such as the liver in later stages of an infection have not been considered in our model. It will be interesting to incorporate data on pDC populations accumulating in peripheral organs during the later phases of MHV infection in future modeling approaches. Likewise, it will be important to address the potential functions of pDCs in the modulation of innate and adaptive immune responses [4] in the terms of systems immunology. A combination of the present mathematical framework with, for example, novel experimental models of pDC-deficiency [28], [29] will open new avenues to describe the dynamical aspects of such multiscale interactions. Furthermore, the presented combination of experimental studies and mathematical modeling may be used to further explore the contribution of virus-encoded factors modulating tumor necrosis factor-enhanced liver inflammation [30] or the role of important host factors such as the prothrombinase Fgl2/fibroleukin which critically regulate virus-induced liver disease [31].

The last decade of research in immunology is characterized by a tremendous advance in the high-throughput experimental technologies yielding detailed information on the system state at various levels of resolution. This inspired a turn in basic and applied immunology from reductionist dissection to systems integration with mathematical modeling being an essential tool [32]–[35]. However, the translation of the powerful modeling methodologies developed in applied mathematics, such as the mathematical systems theory and computational techniques into research tools appropriate for a multiscale analysis of immunological phenomena remains a challenge [1], [36], [37]. Indeed, recent reviews on the application of mathematical analyses in immunology indicate that progress has mainly been made in those studies which model the immune processes at a single resolution level, rather than bridge multiple scales of description [1], [38]–[40]. The within-host population dynamics of antigen-specific immune responses and pathogens and the single cell regulation of lymphocyte activation represent the two most advanced fields. Compared to antigen-specific responses, the development of mathematical models for the description and analysis of innate immune processes has remained a poorly investigated area [41]–[43]. Our study addresses the above two challenges of the integrative modeling of antiviral immune responses: (1) it bridges the cell-cytokine-virus population dynamics level with the physiological function of the spleen in the host-pathogen interaction and (2) provides a high-resolution quantitative description of the early type I IFN response to cytopathic virus infection.

Taken together, the data-driven mathematical modeling of pDC biology provides novel insight in systems' level phenomena such as the pDC protective capacity, the pDC unit of protection, and the sink versus source function of the spleen. Furthermore, this systems immunology approach has generated an in-depth to understanding of the sensitivity of virus-host interaction indicating that antiviral compounds directed against cytopathic viruses should mainly target viral spread within non-lymphoid target organs because pDC-derived type I IFNs within SLOs secure efficient protection of vulnerable target cells.

## Materials and Methods

### Ethics statement

Experiments were performed in accordance with federal and cantonal guidelines (Tierschutzgesetz) under the permission numbers SG07/62 and SG07/63 granted by the Veterinary Office of the Canton of St. Gallen.

### Experimental procedures

C57BL/6 (B6) mice were obtained from Charles River Laboratories (Sulzfeld, Germany). Type I IFN receptor deficient mice (*ifnar^−/−^*) [44] on the B6 background were kindly provided by Martin Bachmann, Cytos AG, Schlieren, Switzerland. MHV A59 was generated from a molecularly cloned cDNA [45] based on the Albany strain of MHV A59 and propagated on L929 cells. EGFP-recombinant MHV was previously described [13]. Mice were sacrificed at the indicated time points and organs were stored at −70°C until further analysis. Blood was incubated at RT to coagulate, centrifuged, and serum was used for ALT measurements using a Hitachi 747 autoanalyzer. Virus titers in organs were determined from frozen organs after weighing and homogenization by standard plaque assay using L929 cells. pDCs were obtained from spleens of B6 mice following digestion with collagenase type II as described previously [8] and infection kinetics following incubation with EGFP-recombinant MHV was determined by flow cytometry [10]. Mφs were isolated from the peritoneal cavity of B6 mice and cell survival was determined with the Cell Proliferation MTS Assay (Celltiter 96 Aqueous one solution cell proliferation assay) from Promega.

### Kinetics of virus, IFN and cells in vitro

The persistence of virus and type I IFN in medium displays an exponential kinetics. The corresponding decay rate constants for MHV (*d_V_*) and IFNα (*d_I_*) were estimated by a linear regression procedure for the log-transformed values of the virus titer and IFN concentration using GraphPad Prism v.4 software (http://www.graphpad.com). The parameter estimates and their 95% Confidence Intervals (CIs) are presented in Table S1.

The data on target cell persistence (*C(t)*) display kinetics which differs for some cell types or under certain conditions from the exponential (denoted by **E**) behavior 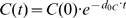. It is rather consistent with the Gompertz kinetics (denoted by **G**) as described by equation
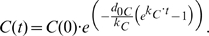



In contrast to the exponential decay, Gompertz kinetics allows the death rate to increase over time and is particularly appropriate for describing cohort-type cell population dynamics. The parameter *k_C_* represents the tempo of the per capita death increase, and for *k_C_* small compared to the duration of experiment, the Gompertz equation reduces to the exponential one. Fitting the cell persistence data using either E-model or G-model showed that

- Mφ survival over the time of the experiment (2 to 3 *days*) is best approximated by the exponential decay with a half-life of 131 (*hours*), whereas
- pDCs follow the Gompertz kinetics with an initial half-life of 125 (*hours*) which reduces by a factor of 8.5 (to 14.7 *hours*) by day 1 of the experiment.

The best-fit estimates for the death rate parameters for pDCs and Mφs are given in the Table S1. To check whether the increased complexity of the G- versus E- model of decay is justified by the pDCs data in hand, we evaluated for the models the Akaike criterion of the information loss (*AIC*), defined as 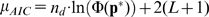 [46], and the model description length [47] evaluated from
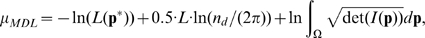
where *n_d_* is the total number of scalar observations, *L* is the number of optimized parameters, 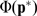 and 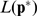 are the best-fit least-squares function and the maximized likelihood function, *I*(**p**) is the Fisher information matrix, and 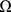 is the domain of the parameter space on which the model is defined. Both criteria of the model parsimony turned out to be smaller for the Gompertz model: the 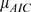 was 30 versus 38 and the 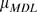 value was 19.5 versus 22.1.

### Basic IFN response modules

To describe the antiviral IFN-α response in vitro, we reduced the complexity of the IFN system to four principal constituents: the virus titer/ml, *V(t)*; the amount of type I IFN per ml, *I(t)*; the density of uninfected target cells (pDC or Mφ), *C(t)*; the density of infected target cells, *C_V_(t)*. The rate of change of the virus population is determined by virus secretion from infected cells, which starts after some latent period (time-delay) 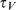, and the elimination through the infection of target cells with the rate constant 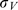 and a natural decay at rate 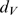. The virus dynamics is modeled by the delay-differential equation:
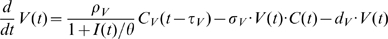
(1)The protective effect of the type I IFN is assumed to reduce the mean per cell virus production rate by 50% at the IFN concentration specified by the inhibition constant *θ*.

The rate of change of the amount of IFN-α in the system results from the IFN production by virus infected target cells, which occurs with some secretion delay, and the decay of the free interferon molecules:
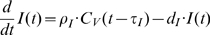
(2)


The parameters of the equation, i.e. the average per cell secretion rate 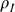, the delay 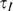 and the decay rate 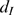 determine the IFN-α concentration dynamics. The loss of IFN-α due to interaction with the target cell receptors is neglected.

The rate of change of the number of infected target cells is modeled by the following equation
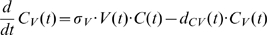
(3)


The first term in the right-hand side describes the emergence of the infected cells due to the virus infection of uninfected target cells whereas the second one accounts for the death of infected cells. The per capita death rate 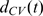 is either constant, or depends on time according to Gompertz law.

Finally, the rate equation for the density of uninfected target cells reads
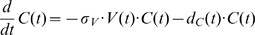
(4)It considers the transition of uninfected cells to infected cells due to virus infection and the death of cells at per capita rate 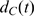, which can be also time-dependent.

### Compartmental model of virus growth

The infection of mice with MHV leads to virus spread and growth in different organs. Virus population dynamics in any anatomical compartment results from a superposition of intra-compartmental production-elimination and inter-compartmental transfer of the virus. The compartmental model in order to describe the pathological consequences of MHV infection requires consideration of the virus population dynamics in spleen 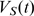, liver 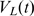 and blood 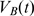. The simplest equations for the rate of change of virus populations in the compartments are as follows:
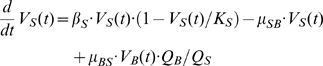
(5)

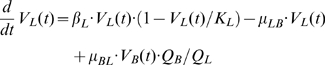
(6)

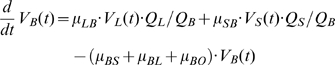
(7)


The virus populations in spleen and liver are assumed to follow a logistic growth with the outflow-inflow rates depending in a linear way on the virus concentration in the corresponding compartments. The parameters 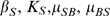 denote the intrinsic growth rate, carrying capacity and transfer rate to and from blood for spleen. Similar parameters characterize the processes in the liver. The blood compartment functions to transfer the virus to spleen, liver and other organs with the rates 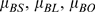, respectively. The intravenous injection of virus dose 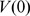 is represented by the following initial conditions: 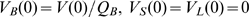. The following estimates of the volumes (*ml*) for spleen, liver and blood were used: 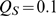, 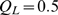, 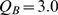 [48].

The infection of target cells with MHV induces a cytopathic effect leading to an earlier cell death. The primary cell targets of MHV in the liver compartment are hepatocytes. The severity of the virus-induced liver disease is characterized by the liver enzyme ALT concentration in serum 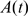. The rate of change of ALT in blood is modeled the following equation:
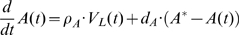
(8)where the increase of serum ALT concentration is proportional to the virus population in the liver, with the parameter 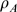 characterizing the release rate of ALT into blood. The second term takes into account that there is some homeostatic turnover of ALT in serum with the decay rate 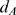.

The compartmental model of virus growth and ALT kinetics provides a tool to quantify the transfer coefficients of the virus for the spleen-blood-liver system (5)–(7) as well as the disease severity due to the virus presence in the liver.

### Systemic model of MHV infection and IFN response

An integrative mathematical model to study the protective function of pDCs in MHV infection is assembled from the building block models described above. The model considers spleen, liver and blood compartments, in which MHV replication is described at different resolution levels. For spleen, a detailed description of the virus-target cells interaction is considered which includes MHV, IFN-α, pDCs, Mφs dynamics as modeled by the subset of equations (9):
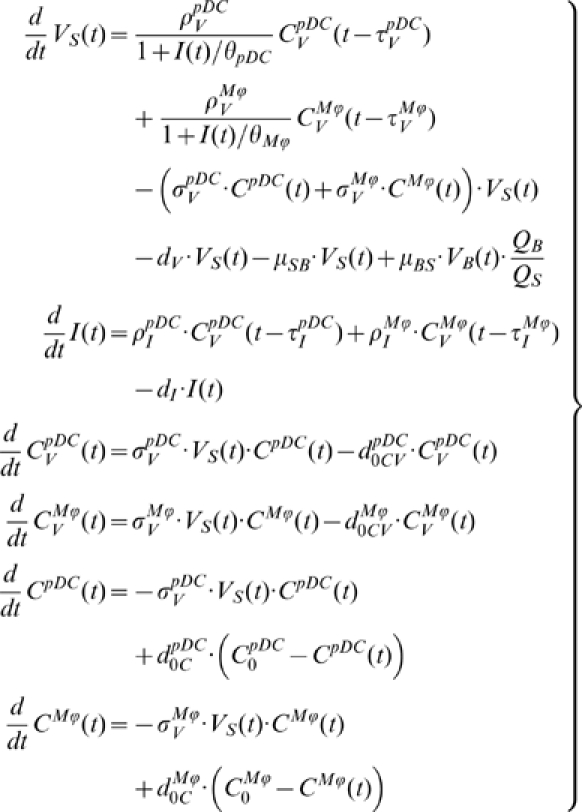
(9)


Eighteen h post MHV infection, spleen contains about 7×10^5^ pDCs and 6×10^6^ Mφs and there is a continuous turnover of these cell populations. Therefore, at any given moment the infected target cells represent a heterogeneous population with respect to the infection time and live expectancy rather than a cohort of cells with synchronous kinetics. This is taken into account by assuming exponential death kinetics for cell populations in spleen, with the rate constants corresponding to the in vitro derived estimates. The influx-elimination processes are described by the homeostasis terms for uninfected pDCs and Mφs (see the last two terms in the last two equations). The equation for virus in spleen considers the virus transfer between spleen and blood. The subset of equations for virus dynamics in blood, liver and the serum ALT level remains the same as equations (6)–(8). The following initial data for the delay differential equation system (6)–(9) specifying the intravenous infection were used
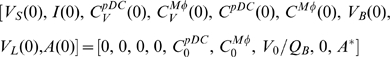
and 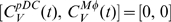 for 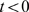. For the numerical solution of the initial value problem for the system of delay-differential equations (6)–(9) we used either the MATLAB code dde23 (http://www.mathworks.com) or our original solver for stiff systems of delay equations [49].

### Parameter estimation and identifiability

The process through which the available information is used in order to estimate as accurately as possible the systems' dynamics is known as data assimilation. To calibrate the mathematical model using the sampled data, we optimally combined heterogeneous observations (coming from distinct series of experiments) with model predictions by optimizing a “cost function”, which expresses the distance between observations and the corresponding model values. The available data vary essentially in terms of the sample sizes, which is common for studies of virus and cell population dynamics. The most numerous data sets are the virus kinetics data. The corresponding samples followed a log-normal distribution. Assuming further that the errors in observations at successive times are independent and the variance of observation errors is constant for all observation times, we applied the maximum likelihood (ML) approach to parameter estimation as we described in detail in [48]. We searched for a vector of best-fit parameters 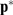, by maximizing the likelihood function 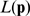 specifying the probability of obtaining the observed data. Under the above assumptions, this optimization is equivalent to minimizing the value of the total misfit between the available observations and the model as defined by 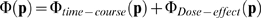. The first term specifies the squared deviation between the log-transformed model and experimental time-series data, and the second one refers (when applicable) to effects of specific treatment (recombinant IFN-α) or infection scenario in vitro (e.g. coculture experiments with pDCs and Mφs) on the virus titer at a given time. The parameter estimation was carried out using either MATLAB 7.0 routines (http://www.mathworks.com) or Absoft Pro Fortran Developer (http://www.absoft.com) and IMSL function minimization code ZXMIN based on a quasi-Newton method.

The number of reliably identifiable parameters in the mathematical models is limited by the amount and quality of the corresponding sets of experimental data which are available and also by the model structure. As the ‘large-scale’ systemic model of MHV infection was built using a modular (rather than monolithic) approach broadly accepted in systems biology [50], we examined the identifiability properties of the basic modules. Having the best-fit parameter estimates and their confidence intervals, we examined a posteriori algebraic identifiability [51] of the modules following the multiple time points method [52]. The method is based upon elimination of unobserved state variable from the original system of equations to obtain the identification equation by a combination of high-order derivatives of the observed variables and the availability of the measurements at a number of time points.

The algebraic identifiability analysis of the basic IFN response module (equations 1–4) showed that if virus titer, IFN concentration and the fraction of live cells are the observable state characteristics, then the time-lag of virus production, the 50% inhibition threshold and the virus secretion rate represent a group of functionally related parameters, i.e. their estimates depend on each other with the statistical uncertainty further to be characterized by the confidence intervals. This dependence is consistent with them entering together the virus production term in equation (1) of the basic module. The other implication is that the structure permits the other model parameters to be identified. The identifiability test of the compartmental model (equations 5–8) suggests that it is identifiable, provided that the observable variables are the virus in spleen, blood and liver and the serum ALT blood, which is the current case. Furthermore, a global sensitivity analysis was performed which allowed to rank the influence of random variations in the model parameters on the variation in the serum ALT level. The methodology and the results are presented in Table S2 and the accompanying text.

## Supporting Information

Figure S1 Viral replication in MHV-infected pDCs in vitro at MOI = 0.1 and 0.01. Virus produced at 24 hours after MHV infection of 10^5^ pDCs predicted by the mathematical model is compared to experimentally observed values. pDCs from wt and *ifnar^−/−^* mice. At MOI = 0.1 (black solid lines, squares) the data represent the geometric mean ± SD from 5 experiments. At MOI = 0.01 (red dotted lines, circles) the data represent the geometric mean ± SD from 2–4 experiments. Taking into account that the variability between experimental series is about 0.5 at the log_10_ - scale, the model calibrated on independent sets of data provides a valid description of the system.
(0.07 MB DOC)

Figure S2 The kinetics of viral replication in wt macrophages in vitro at MOI = 0.0001. The virus (red line), type I IFN (green line) and infected cell (blue line) kinetics are shown. The measurements of the virus titer (circles) observed at 12 and 24 hours after MHV infection of 10^5^ macrophages are depicted.
(0.06 MB DOC)

Figure S3 The effect of pDCs derived supernatant on MHV replication in wt macrophages in vitro at MOI = 1. The virus production in vitro was measured in wt macrophages pre-treated with the indicated amounts of IFNα-containing supernatant (500, 200, 50, 10 pg/ml). Macrophages from B6 mice were plated at 5×10^5^ cells/well of the volume 500 µl. The data represent the geometric mean ± SD from 4 to 8 independent experiments.
(0.05 MB DOC)

Figure S4 Dynamics of MHV infection as described by the ‘in vitro spleen’ approach. The virus kinetics predicted by the mathematical model for in vitro infection of 7×10^5^ pDCs and 6×10^6^ Mφs. Low (5×10^1^ pfu), intermediate- (5×10^3^ pfu) and high (5×10^5^ pfu) dose infections are considered. The model consistently predicts the efficient containment of virus replication (left panel) and the dose dependent activation of the type I IFN synthesis (right panel).
(0.13 MB DOC)

Figure S5 Infection of macrophages assessed by in situ analysis. C57BL/6 mice were infected i.p. with 5×10^5^ pfu MHV. For depletion of pDCs, mice were injected i.p. with 0.5 mg of α-mPDCA-1 (Miltenyi Biotec) 12 h prior to infection or left untreated (n = 3 mice per group). (A) Fluorescence microscopic analysis of spleen sections at 48 h post infection using antibodies against B220 (blue), MHV-N (green) and F4/80 (red). Original magnification (×400). (B) Quantitative evaluation of macrophage infection. Values indicate numbers of MHV-N^+^F4/80^+^ per high power field (mean ± SEM). Three sections from each mouse were analyzed.
(0.25 MB DOC)

Table S1 (0.05 MB DOC)

Table S2 (0.10 MB DOC)

## References

[1] Young D, Stark J, Kirschner D (2008). Systems biology of persistent infection: tuberculosis as a case study.. Nat Rev Microbiol.

[2] Junt T, Scandella E, Ludewig B (2008). Form follows function: lymphoid tissue microarchitecture in antimicrobial immune defence.. Nat Rev Immunol.

[3] Colonna M, Trinchieri G, Liu YJ (2004). Plasmacytoid dendritic cells in immunity.. Nat Immunol.

[4] Villadangos JA, Young L (2008). Antigen-presentation properties of plasmacytoid dendritic cells.. Immunity.

[5] Barchet W, Cella M, Colonna M (2005). Plasmacytoid dendritic cells–virus experts of innate immunity.. Semin Immunol.

[6] Bergmann CC, Lane TE, Stohlman SA (2006). Coronavirus infection of the central nervous system: host-virus stand-off.. Nat Rev Microbiol.

[7] Perlman S, Netland J (2009). Coronaviruses post-SARS: update on replication and pathogenesis.. Nat Rev Microbiol.

[8] Cervantes-Barragan L, Zust R, Weber F, Spiegel M, Lang KS (2007). Control of coronavirus infection through plasmacytoid dendritic-cell-derived type I interferon.. Blood.

[9] Lang PA, Cervantes-Barragan L, Verschoor A, Navarini AA, Recher M (2009). Hematopoietic cell-derived interferon controls viral replication and virus-induced disease.. Blood.

[10] Cervantes-Barragan L, Kalinke U, Zust R, Konig M, Reizis B (2009). Type I IFN-mediated protection of macrophages and dendritic cells secures control of murine coronavirus infection.. J Immunol.

[11] Bocharov G, Ford NJ, Ludewig B (2005). A mathematical approach for optimizing dendritic cell-based immunotherapy.. Methods Mol Med.

[12] Ludewig B, Bocharov G, Lutz M, Romani N, Steinkasserer A (2006). A systems biologist's view on dendritic cell-cytotoxic T lymphocyte interaction.. Handbook of Dendritic Cells. Biology, Diseases and Therapy..

[13] Züst R, Cervantes-Barragan L, Kuri T, Blakqori G, Weber F (2007). Identification of Coronavirus Non-Structural Protein 1 as a Major Pathogenicity Factor - Implications for the Rational Design of Live Attenuated Coronavirus Vaccines.. PLoS Pathog.

[14] Thiel V, Weber F (2008). Interferon and cytokine responses to SARS-coronavirus infection.. Cytokine Growth Factor Rev.

[15] Tumpey TM, Basler CF, Aguilar PV, Zeng H, Solorzano A (2005). Characterization of the reconstructed 1918 Spanish influenza pandemic virus.. Science.

[16] Rolling T, Koerner I, Zimmermann P, Holz K, Haller O (2009). Adaptive mutations resulting in enhanced polymerase activity contribute to high virulence of influenza A virus in mice.. J Virol.

[17] Perlman S, Dandekar AA (2005). Immunopathogenesis of coronavirus infections: implications for SARS.. Nat Rev Immunol.

[18] Junt T, Moseman EA, Iannacone M, Massberg S, Lang PA (2007). Subcapsular sinus macrophages in lymph nodes clear lymph-borne viruses and present them to antiviral B cells.. Nature.

[19] Ochsenbein AF, Pinschewer DD, Odermatt B, Carroll MC, Hengartner H (1999). Protective T cell-independent antiviral antibody responses are dependent on complement.. J Exp Med.

[20] de Lang A, Baas T, Smits SL, Katze MG, Osterhaus AD (2009). Unraveling the complexities of the interferon response during SARS-CoV infection.. Future Virol.

[21] Phipps-Yonas H, Seto J, Sealfon SC, Moran TM, Fernandez-Sesma A (2008). Interferon-beta pretreatment of conventional and plasmacytoid human dendritic cells enhances their activation by influenza virus.. PLoS Pathog.

[22] Shortman K, Naik SH (2007). Steady-state and inflammatory dendritic-cell development.. Nat Rev Immunol.

[23] Wollenberg A, Wagner M, Gunther S, Towarowski A, Tuma E (2002). Plasmacytoid dendritic cells: a new cutaneous dendritic cell subset with distinct role in inflammatory skin diseases.. J Invest Dermatol.

[24] de Heer HJ, Hammad H, Soullie T, Hijdra D, Vos N (2004). Essential role of lung plasmacytoid dendritic cells in preventing asthmatic reactions to harmless inhaled antigen.. J Exp Med.

[25] Wendland M, Czeloth N, Mach N, Malissen B, Kremmer E (2007). CCR9 is a homing receptor for plasmacytoid dendritic cells to the small intestine.. Proc Natl Acad Sci U S A.

[26] Cella M, Jarrossay D, Facchetti F, Alebardi O, Nakajima H (1999). Plasmacytoid monocytes migrate to inflamed lymph nodes and produce large amounts of type I interferon.. Nat Med.

[27] Yoneyama H, Matsuno K, Zhang Y, Nishiwaki T, Kitabatake M (2004). Evidence for recruitment of plasmacytoid dendritic cell precursors to inflamed lymph nodes through high endothelial venules.. Int Immunol.

[28] Cisse B, Caton ML, Lehner M, Maeda T, Scheu S (2008). Transcription factor E2-2 is an essential and specific regulator of plasmacytoid dendritic cell development.. Cell.

[29] Swiecki M, Colonna M (2010). Unraveling the functions of plasmacytoid dendritic cells during viral infections, autoimmunity, and tolerance.. Immunol Rev.

[30] Eriksson KK, Cervantes-Barragan L, Ludewig B, Thiel V (2008). Mouse hepatitis virus liver pathology is dependent on ADP-ribose-1″-phosphatase, a viral function conserved in the alpha-like supergroup.. J Virol.

[31] Marsden PA, Ning Q, Fung LS, Luo X, Chen Y (2003). The Fgl2/fibroleukin prothrombinase contributes to immunologically mediated thrombosis in experimental and human viral hepatitis.. J Clin Invest.

[32] Germain RN (2001). The art of the probable: system control in the adaptive immune system.. Science.

[33] Tan SL, Ganji G, Paeper B, Proll S, Katze MG (2007). Systems biology and the host response to viral infection.. Nat Biotechnol.

[34] Gardy JL, Lynn DJ, Brinkman FS, Hancock RE (2009). Enabling a systems biology approach to immunology: focus on innate immunity.. Trends Immunol.

[35] Zak DE, Aderem A (2009). Systems biology of innate immunity.. Immunol Rev.

[36] Kirschner DE, Linderman JJ (2009). Mathematical and computational approaches can complement experimental studies of host-pathogen interactions.. Cell Microbiol.

[37] Meier-Schellersheim M, Fraser IDC, Klauschen F (2009). Multiscale modeling for biologists.. WIREs Systems Biology and Medicine.

[38] Perelson AS (2002). Modelling viral and immune system dynamics.. Nat Rev Immunol.

[39] Antia R, Ganusov VV, Ahmed R (2005). The role of models in understanding CD8+ T-cell memory.. Nat Rev Immunol.

[40] Beltman JB, Maree AF, De Boer RJ (2009). Analysing immune cell migration.. Nat Rev Immunol.

[41] Bocharov GA, Romanyukha AA (1994). Mathematical model of antiviral immune response. III. Influenza A virus infection.. J Theor Biol.

[42] Howat TJ, Barreca C, O'Hare P, Gog JR, Grenfell BT (2006). Modelling dynamics of the type I interferon response to in vitro viral infection.. J R Soc Interface.

[43] Tegner J, Nilsson R, Bajic VB, Bjorkegren J, Ravasi T (2006). Systems biology of innate immunity.. Cell Immunol.

[44] Muller U, Steinhoff U, Reis LF, Hemmi S, Pavlovic J (1994). Functional role of type I and type II interferons in antiviral defense.. Science.

[45] Coley SE, Lavi E, Sawicki SG, Fu L, Schelle B (2005). Recombinant mouse hepatitis virus strain A59 from cloned, full-length cDNA replicates to high titers in vitro and is fully pathogenic in vivo.. J Virol.

[46] Johnson JB, Omland KS (2004). Model selection in ecology and evolution.. Trends Ecol Evol.

[47] Pitt MA, Myung IJ (2002). When a good fit can be bad.. Trends Cogn Sci.

[48] Ludewig B, Krebs P, Junt T, Metters H, Ford NJ (2004). Determining control parameters for dendritic cell-cytotoxic T lymphocyte interaction.. Eur J Immunol.

[49] Bocharov G, Marchuk GI, Romanyukha AA (1996). Numerical solution by LMMs of stiff delay differential systems modelling an immune response.. Numerische Mathematik.

[50] van Riel NA (2006). Dynamic modelling and analysis of biochemical networks: mechanism-based models and model-based experiments.. Brief Bioinform.

[51] Xia X, Moog CH (2003). Identifiability of nonlinear systems with applications to HIV/AIDS models.. IEEE Transaction of Automatic Control.

[52] Wu H, Zhu H, Miao H, Perelson AS (2008). Parameter identifiability and estimation of HIV/AIDS dynamic models.. Bull Math Biol.

