# Supplementary material for: A Systems Immunology Approach to Plasmacytoid Dendritic Cell Function in Cytopathic Virus Infections
Source: PLoS Pathog. 2010 Jul 22;6(7):e1001017. doi: 10.1371/journal.ppat.1001017 (PMC2908624; doi:10.1371/journal.ppat.1001017)
Supplement: Figure S1 — Viral replication in MHV-infected pDCs in vitro at MOI = 0.1 and 0.01. Virus produced at 24 hours after MHV infection of 105 pDCs predicted by the mathematical model is compared to experimentally observed values. pDCs from wt and ifnar−/− mice. At MOI = 0.1 (black solid lines, squares) the data represent the geometric mean ± SD from 5 experiments. At MOI = 0.01 (red dotted lines, circles) the data represent the geometric mean ± SD from 2–4 experiments. Taking into account that the variability between experimental series is about 0.5 at the log10 - scale, the model calibrated on independent sets of data provides a valid description of the system. (0.07 MB DOC) [file ppat.1001017.s001.doc]

**Supporting information figure 1. Viral replication in MHV-infected pDCs in vitro at MOI = 0.1 and 0.01.** Virus produced at 24 hours after MHV infection of 10^5^ pDCs predicted by the mathematical model is compared to experimentally observed values. pDCs from wt and *ifnar^-/-^* mice. At MOI = 0.1 (black solid lines, squares) the data represent the geometric mean ± SD from 5 experiments. At MOI = 0.01 (red dotted lines, circles) the data represent the geometric mean ± SD from 2 – 4 experiments. Taking into account that the variability between experimental series is about 0.5 at the log_10_ - scale, the model calibrated on independent sets of data provides a valid description of the system.
