# Supplementary material for: A Systems Immunology Approach to Plasmacytoid Dendritic Cell Function in Cytopathic Virus Infections
Source: PLoS Pathog. 2010 Jul 22;6(7):e1001017. doi: 10.1371/journal.ppat.1001017 (PMC2908624; doi:10.1371/journal.ppat.1001017)
Supplement: Figure S2 — The kinetics of viral replication in wt macrophages in vitro at MOI = 0.0001. The virus (red line), type I IFN (green line) and infected cell (blue line) kinetics are shown. The measurements of the virus titer (circles) observed at 12 and 24 hours after MHV infection of 105 macrophages are depicted. (0.06 MB DOC) [file ppat.1001017.s002.doc]

**Supporting information figure 2. The kinetics of viral replication in wt macrophages in vitro at MOI = 0.0001.** The virus (red line), type I IFN (green line) and infected cell (blue line) kinetics are shown. The measurements of the virus titer (circles) observed at 12 and 24 hours after MHV infection of 10^5^ macrophages are depicted.
