# Supplementary material for: A Systems Immunology Approach to Plasmacytoid Dendritic Cell Function in Cytopathic Virus Infections
Source: PLoS Pathog. 2010 Jul 22;6(7):e1001017. doi: 10.1371/journal.ppat.1001017 (PMC2908624; doi:10.1371/journal.ppat.1001017)
Supplement: Figure S3 — The effect of pDCs derived supernatant on MHV replication in wt macrophages in vitro at MOI = 1. The virus production in vitro was measured in wt macrophages pre-treated with the indicated amounts of IFNα-containing supernatant (500, 200, 50, 10 pg/ml). Macrophages from B6 mice were plated at 5×10^5^ cells/well of the volume 500 µl. The data represent the geometric mean ± SD from 4 to 8 independent experiments. (0.05 MB DOC) [file ppat.1001017.s003.doc]

**Supporting information figure 3. The effect of pDCs derived supernatant on MHV replication in wt macrophages in vitro at MOI = 1.** The virus production in vitro was measured in wt macrophages pre-treated with the indicated amounts of IFNα-containing supernatant (500, 200, 50, 10 pg/ml). Macrophages from B6 mice were plated at 5x10^5^ cells/well of the volume 500 μl. The data represent the geometric mean ± SD from 4 to 8 independent experiments.
