# Supplementary material for: A Systems Immunology Approach to Plasmacytoid Dendritic Cell Function in Cytopathic Virus Infections
Source: PLoS Pathog. 2010 Jul 22;6(7):e1001017. doi: 10.1371/journal.ppat.1001017 (PMC2908624; doi:10.1371/journal.ppat.1001017)
Supplement: Figure S4 — Dynamics of MHV infection as described by the ‘in vitro spleen’ approach. The virus kinetics predicted by the mathematical model for in vitro infection of 7×10^5^ pDCs and 6×10^6^ Mφs. Low (5×101 pfu), intermediate- (5×10^3^ pfu) and high (5×10^5^ pfu) dose infections are considered. The model consistently predicts the efficient containment of virus replication (left panel) and the dose dependent activation of the type I IFN synthesis (right panel). (0.13 MB DOC) [file ppat.1001017.s004.doc]

**
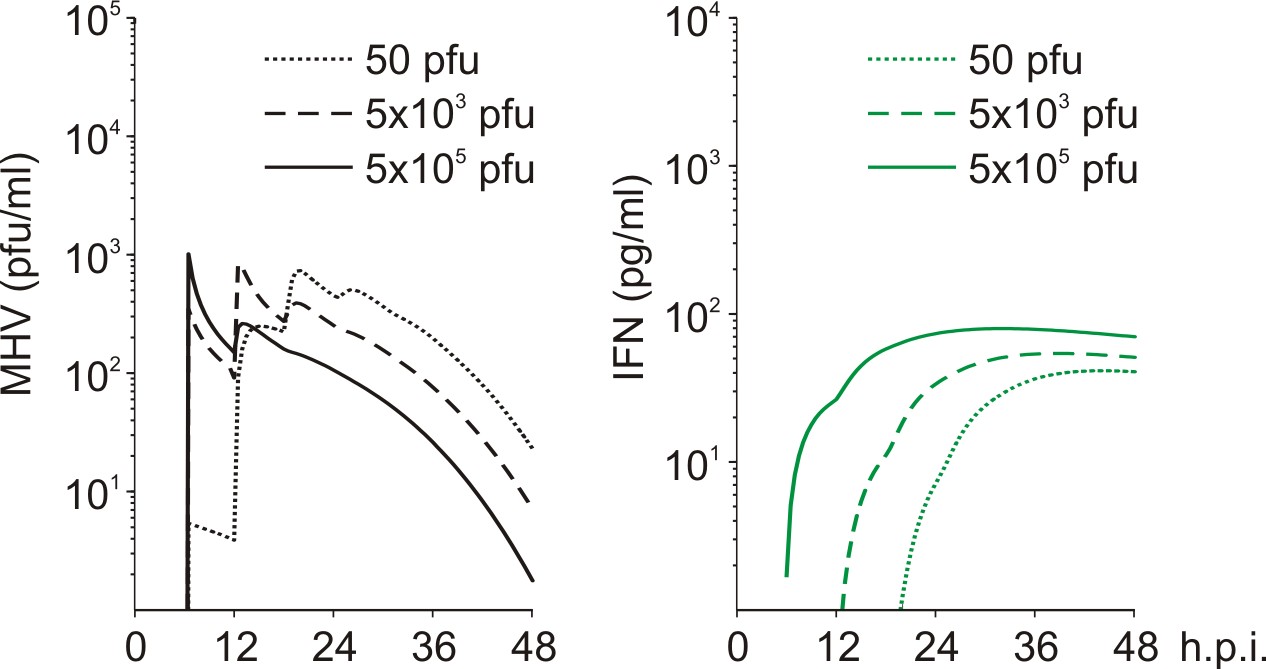
**

**Supporting information figure 4. Dynamics of MHV infection as described by the ‘in vitro spleen’ approach.** The virus kinetics predicted by the mathematical model for in vitro infection of 7×10^5^ pDCs and 6×10^6^ Mφs. Low (5×10^1^ pfu), intermediate- (5×10^3^ pfu) and high (5×10^5^ pfu) dose infections are considered. The model consistently predicts the efficient containment of virus replication (left panel) and the dose dependent activation of the type I IFN synthesis (right panel).
