# Supplementary material for: A Systems Immunology Approach to Plasmacytoid Dendritic Cell Function in Cytopathic Virus Infections
Source: PLoS Pathog. 2010 Jul 22;6(7):e1001017. doi: 10.1371/journal.ppat.1001017 (PMC2908624; doi:10.1371/journal.ppat.1001017)
Supplement: Figure S5 — Infection of macrophages assessed by in situ analysis. C57BL/6 mice were infected i.p. with 5×10^5^ pfu MHV. For depletion of pDCs, mice were injected i.p. with 0.5 mg of α-mPDCA-1 (Miltenyi Biotec) 12 h prior to infection or left untreated (n = 3 mice per group). (A) Fluorescence microscopic analysis of spleen sections at 48 h post infection using antibodies against B220 (blue), MHV-N (green) and F4/80 (red). Original magnification (×400). (B) Quantitative evaluation of macrophage infection. Values indicate numbers of MHV-N+F4/80+ per high power field (mean ± SEM). Three sections from each mouse were analyzed. (0.25 MB DOC) [file ppat.1001017.s005.doc]

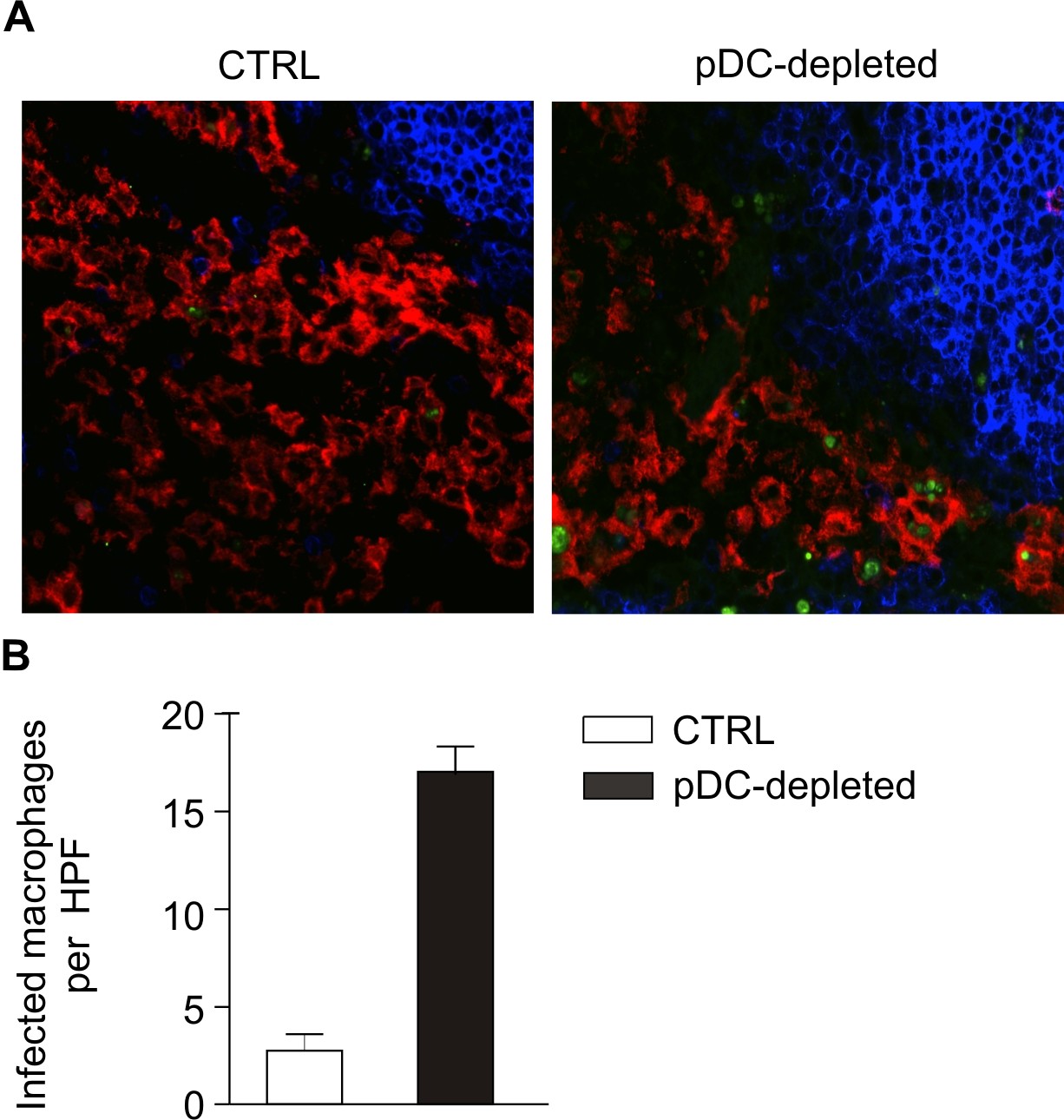


**Supporting information figure 5. Infection of macrophages assessed by in situ analysis.** C57BL/6 mice were infected i.p. with 5×10^5^ pfu MHV. For depletion of pDCs, mice were injected i.p. with 0.5 mg of α-mPDCA-1 (Miltenyi Biotec) 12 h prior to infection or left untreated (n=3 mice per group). (A) Fluorescence microscopic analysis of spleen sections at 48 h post infection using antibodies against B220 (blue), MHV-N (green) and F4/80 (red). Original magnification (×400). (B) Quantitative evaluation of macrophage infection. Values indicate numbers of MHV-N^+^F4/80^+^ per high power field (mean ± SEM). Three sections from each mouse were analyzed.
