# Supplementary material for: A Systems Immunology Approach to Plasmacytoid Dendritic Cell Function in Cytopathic Virus Infections
Source: PLoS Pathog. 2010 Jul 22;6(7):e1001017. doi: 10.1371/journal.ppat.1001017 (PMC2908624; doi:10.1371/journal.ppat.1001017)
Supplement: Table S1 — (0.05 MB DOC) [file ppat.1001017.s006.doc]

**Supporting information table 1. Estimated decay- or death rates and their 95% confidence intervals (CI) for MHV, IFNα, pDC and Mφ**

| **Parameter** | **Biological meaning** (units) | **Best-fit estimate [95%** **CI]** |
| --- | --- | --- |
| *d_V_* | MHV decay rate constant in medium (*1/h*) | 0.155; [0.12, 0.19]  ^(*)^ 0.12 used for in vivo data |
| *d_I_* | IFNα decay rate constant in medium (*1/h*) | 0.012; [0.0076, 0.016] |
| pDC: *d_0C_*  *k_C_* | Uninfected cell death rate constants, G-model: (*1/h*) | 0.0055; [0.00038, 0.011],  0.089; [0.044, 0.13] |
| Mφ: *d_0C_* | Uninfected cell death rate constant, E-model: (*1/h*) | 0.0053; [0.0016, 0.0090] |
