# Supplementary material for: A Systems Immunology Approach to Plasmacytoid Dendritic Cell Function in Cytopathic Virus Infections
Source: PLoS Pathog. 2010 Jul 22;6(7):e1001017. doi: 10.1371/journal.ppat.1001017 (PMC2908624; doi:10.1371/journal.ppat.1001017)
Supplement: Table S2 — (0.10 MB DOC) [file ppat.1001017.s007.doc]

**Supporting information table 2.** The analysis of correlation coefficients between the model parameters and the serum ALT at 48 hours after infection for low and intermediate dose i.v. infections

| **Parameter of the model** | **Correlation Coefficient (dose of infection 50 pfu)** | **Correlation Coefficient (dose of infection 5000 pfu)** |
| --- | --- | --- |
| $\rho_{V}^{pDC}$ | 0.0023 | 0.011 |
| $\rho_{V}^{M\varphi}$ | 0.19 | 0.17 |
| $\rho_{I}^{pDC}$ | -0.13 | -0.12 |
| $\rho_{I}^{M\varphi}$ | -0.049 | -0.055 |
| $\theta_{pDC}$ | 0.014 | -0.0025 |
| $\theta_{M\varphi}$ | 0.23 | 0.20 |
| $\sigma_{V}^{pDC}$ | -0.071 | -0.054 |
| $\sigma_{V}^{M\varphi}$ | 0.089 | 0.061 |
| $d_{0CV}^{pDC}$ | 0.16 | 0.18 |
| $d_{0CV}^{M\varphi}$ | -0.0051 | -0.017 |
| $d_{0C}^{pDC}$ | 0.0042 | -0.0065 |
| $d_{0C}^{M\varphi}$ | 0.0046 | -0.000029 |
| $d_{V}$ | -0.0049 | 0.00072 |
| $d_{I}$ | -0.0024 | -0.00057 |
| $\mu_{SB}$ | 0.072 | 0.065 |
| $\mu_{BS}$ | -0.061 | -0.082 |
| $\beta_{L}$ | 0.75 | 0.75 |
| $K_{L}$ | 0.17 | 0.18 |
| $\mu_{LB}$ | -0.79 | -0.80 |
| $\mu_{BL}$ | 0.15 | 0.18 |
| $\rho_{A}$ | 0.41 | 0.41 |
| $d_{A}$ | -0.32 | -0.32 |
| $\mu_{B0}$ | -0.11 | -0.083 |
| $\tau_{V}^{pDC}$ | 0.014 | -0.0032 |
| $\tau_{V}^{M\varphi}$ | 0.00092 | -0.0071 |
| $\tau_{I}^{pDC}$ | 0.0121 | 0.0056 |
| $\tau_{I}^{M\varphi}$ | 0.0061 | -0.0061 |

It is widely accepted that a typical problem with developing mechanistic mathematical models of immune processes is that part of the model parameters appear to be rather uncertain. The number of reliably identifiable parameters in the mathematical models is limited by the amount and quality of the corresponding sets of experimental data which are available and also by the model structure which usually has no a priori proof of validity. We applied the global sensitivity analysis to rank the model parameters with respect to their impact on the model solution. As an integrative model output (outcome variable) we considered the serum ALT level at 48 hours after infection The sensitivity analysis was performed using sampling-based sensitivity index partial rank correlation coefficient (PRCC) (using the code available at <http://malthus.micro.med.umich.edu/lab/usadata/>) combined with Latin Hypercube Sampling (LHS) method. Calculation of PRCC enables the determination of the statistical relationships between each model parameter (input variable) and the specified outcome variable with the sign indicating the qualitative relationship and the magnitude quantifying the effect of uncertainty in the parameter value in contributing to the imprecision in predicting the model output value. The LHS was performed using triangle probability density functions for the model parameters with the peak value being the best-fit estimate. The ranges were taken to be the estimated confidence intervals for the best-fit parameter values. The sample sizes were 10^4^. The correlation coefficients computed for i.v. MHV infection with 50 and 5000 pfu are presented in Table 2 above. The most critical parameters are those characterizing the infection in the liver. Among the splenic parameters the virus synthesis rate by macrophages, the sensitivity of macrophages to type I IFN and the IFN secretion rate by pDCs have the highest effect on the disease severity.
